# “Every breath you take”: evaluating sound levels and acoustic characteristics of various neonatal respiratory support and ventilation modalities

**DOI:** 10.3389/fped.2024.1379249

**Published:** 2024-04-19

**Authors:** Sophie Stummer, Christoph Reuter, Isabella Czedik-Eysenberg, Matthias Bertsch, Katrin Klebermass-Schrehof, Johannes Mader, Julia Buchmayer, Lisa Bartha-Doering, Angelika Berger, Vito Giordano

**Affiliations:** ^1^ Department of Pediatrics and Adolescent Medicine, Division of Neonatology, Pediatric Intensive Care and Neuropediatrics, Comprehensive Center for Pediatrics, Medical University of Vienna, Vienna, Austria; ^2^ Department of Pediatrics and Adolescent Medicine, Division of Pediatric Pulmonology, Allergology and Endocrinology, Medical University of Vienna, Vienna, Austria; ^3^ Musicological Department, University of Vienna, Vienna, Austria; ^4^ Department of Music Physiology, University of Music and Performing Arts Vienna, Vienna, Austria

**Keywords:** NICU, noise, preterm infants, CPAP, HFNC, HFOV

## Abstract

**Background:**

Early sensory experiences have a significant impact on the later life of preterm infants. The NICU soundscape is profoundly influenced by various modalities of respiratory support or ventilation, which are often mandatory early in the care. The incubator, believed to shield from external noise, is less effective against noise originating inside. The objective of this study was to evaluate the sound levels and characteristics of frequently used respiratory support and ventilation modalities, taking into consideration the developing auditory system of premature infants.

**Methods:**

To evaluate sound dynamics inside and outside an incubator during respiratory support/ventilation, experimental recordings were conducted at the Center for Pediatric Simulation Training of the Medical University Vienna. The ventilator used was a FABIAN HFOI®.

**Results:**

Jet CPAP (Continuous positive airway pressure), whether administered via mask or prongs, generates significantly higher sound levels compared to High-flow nasal cannula (HFNC) and to High-frequency oscillatory ventilation (HFOV) delivered through an endotracheal tube. Upon evaluating the sound spectrum of jet CPAP support, a spectral peak is observed within the frequency range of 4 to 8 kHz. Notably, this frequency band aligns with the range where the hearing threshold of preterm infants is at its most sensitive.

**Conclusion:**

Non-invasive HFNC and invasive HFOV generate lower sound levels compared to those produced by jet CPAP systems delivered via masks or prongs. Moreover, HFNC and HFOV show a reduced acoustic presence within the frequency range where the preterm infant’s hearing is highly sensitive. Therefore, it is reasonable to speculate that the potential for auditory impairment might be more pronounced in preterm infants who require prolonged use of jet CPAP therapy during their time in the incubator.

## Introduction

Infants born before 37 weeks of gestation are defined as preterm infants. According to the World Health Organization (WHO), more than 10 million infants are affected every year (1).

The constant improvement of neonatal intensive care leads to an increasing number of surviving preterm infants (2). However, preterm birth contributes to an impaired brain maturation and thus, can lead to impaired neurocognitive outcome. Especially infants born extremely preterm are at risk of visual, hearing or developmental impairments (3).

Perinatal sensory experiences affect later life of premature infants, as the transition from intra- to extrauterine life is considered a particularly vulnerable period for these infants. During this phase, they must adapt to a markedly different extrauterine environment (4, 5).

The structural development of the auditory system starts early in week 15 of gestation, the functional development follows at weeks 23–29. Nowadays, active management of preterm infants starts as early as 22 weeks of gestation. Even though the auditory system is not fully developed at this stage, there is evidence that preterm infants as young as 24 weeks can actively respond to auditory stimuli (6, 7).

The auditory system needs external stimulation during fetal life, including voices and language, music, and various environmental sounds, in order to develop properly (8, 9). Therefore, external stimuli are crucial not only *in utero* but also in the neonatal intensive care unit (NICU) in case of preterm birth, as they significantly influence auditory outcomes (10). However, preterm infants are exposed to an unphysiological soundscape in the NICU. In the maternal womb, extrauterine sounds are filtered by the mother's body and the amniotic fluid, leading to an attenuation of sounds most times not exceeding 30 dB, and it is mainly low frequency sounds that are being transmitted (11). In contrast, the soundscape in the NICU consists primarily of various high frequency noises exceeding recommended noise levels for neonates by far (12–15).

According to the American Academy for Pediatrics (AAP), the noise level in NICUs shall not exceed 45 dB during daytime and 35 dB at night. The high noise levels common in NICUs (12–14) might lead to adverse outcomes regarding both the acoustic as well as the neurological development of preterm infants.

Preterm infants are at higher risk of experiencing environmentally induced hearing loss. While less than 5% suffer from complete deafness, up to 50% exhibit signs of acoustic trauma (16–18). Moreover, sustained exposure to a soundscape outside the decibel and frequency range physiologically heard by the fetus has also been related to stress responses, altered physiological stability, sleep deprivation and alterations in the autonomic, metabolic, and endocrine systems of preterm infants (19).

In the NICU, the incubator provides some protection of the premature infant against external stimuli (20, 21). While there is a reduction of sound transmission from outside the incubator, less is known about the sound dynamics and sound characteristics of noises generated inside the incubator, primarily from medical devices such as those used for respiratory support (20).

Bertsch et al. (20), and Reuter et al. (21), further reported the incubator to be a bass booster for noises generated outside the incubator, while Kaiser et al. (22), emphasizes the high frequency range produced by non-invasive ventilation within the incubator.

Considering this, the aim of this study was to gather deeper insights into the specific dynamics and characteristics of sounds produced by devices typically used for respiratory support and ventilation, both inside and outside the incubator.

## Materials and methods

### Setting

This study was conducted at the Pediatric Simulation Centre of the Medical University of Vienna, Comprehensive Center for Pediatrics. To evaluate the noise levels of various types of respiratory support and ventilation, measurements were taken inside and outside the incubator.

### Material

Audio recordings of four common types of respiratory support and invasive ventilation, respectively, were measured on a simulation manikin (Premature Anne®, Laerdal) lying on a padded heating mat in a Dräger Babyleo TN 500® incubator. Premature Anne® authentically represents a preterm infant of 25 weeks of gestation—including an exact replica of airway and body dimensions. The incubator has a dimension of 1,154 mm width and 690 mm depth. The simulation room measured 6,500 mm × 3,500 mm with a height of 2,400 mm. The ventilator used was a FABIAN HFOI®.

This device, specifically designed for neonatal and pediatric care, is known to be very versatile, an all-in-one therapy system that can offer various modes of ventilation and respiratory support: (1) continuous positive airway pressure (CPAP); (2) high-flow nasal cannula (HFNC); (3) synchronized intermittent mandatory ventilation (SIMV); (4) pressure support ventilation (PSV); (5) volume guarantee (VG) ventilation; (6) non-invasive ventilation (NIV) modes (gentle ventilation strategies that do not require intubation, including bi-level positive airway pressure (BiPAP) or variable positive airway pressure (VPAP), providing alternating levels of pressure to assist with the infant's breathing cycle).

Regarding the CPAP, the equipment including tubing system as well as mask and prongs of Infant Flow® was used. The Infant Flow® patented dual-jet variable flow generator utilizes fluidic technology to deliver a constant CPAP at the airway proximal to the infant's nares.

The recordings were made with two Esper K4 measurement microphones (calibrated to 114 dB_SPL_ at 1,000 Hz), one at the ear of the manikin inside the incubator (57 cm below the incubator ceiling) and one on the same vertical axis outside the incubator (57 cm above the incubator). An additional matching level measurement was made using an NTi XL2 Acoustic Analyser. Furthermore, an acoustic camera (Gfai Mikado 96 microphones array) was used to visualize the generation and propagation of sound.

### Measurements

The following modes of respiratory support/ventilation were measured:

- High-flow nasal cannula (HFNC) at flow rates of 2, 4, 6, 8, 10 and 12 L/min.

- Jet CPAP via mask at PEEP levels of 2, 4, 6, 8, 10 and 12 mbar.

- Jet CPAP via nasal prongs at PEEP levels of 2, 4, 6, 8, 10 and 12 mbar.

- High-frequency oscillatory ventilation (HFOV) via endotracheal tube at MAP levels of 8, 12 and 15 mbar and an amplitude of 20, 40 and 60 mbar.

Measurements of all recording conditions were taken using Esper K4 measurement microphones. These 1/4-inch omnidirectional free-field microphones have a frequency response 20–25,000 Hz with a tolerance of ±2 dB. Recordings were made both inside and outside the incubator in the Pediatric Simulation Centre.

For each measurement in non-invasive respiratory support, the intensity was increased by 2 units (either in L/min or mbar, depending on whether HFNC or CPAP was used).

### Sound analysis

Third-octave spectral analysis of the recorded data as well as signal analysis in regards to timbral characteristics were performed using MIRtoolbox in Matlab (23), Librosa (24) and the AudioCommons Timbral Models (25) in Python. The acoustic camera images were calculated using Gfai NoiseImage. Statistical analysis was performed using JASP (JASP Team. JASP (Version 0.17.2). Amsterdam (2023). https://jasp-stats.org). Normal distribution was tested using the Shapiro–Wilk-test, and data were visualized with Plotly [Plotly Technologies Inc. Collaborative data science. Montréal, QC, (2015). https://plot.ly].

## Results

### Non-invasive respiratory support

Our measurements indicated that noise levels of non-invasive respiratory support devices consistently surpassed 70 dB_SPL_ inside the incubator. The noise level generated by jet CPAP was significantly higher than that generated by HFNC, regardless of whether prongs or a mask were used. There was a strong increase in noise levels across all modes of respiratory support as the level of support intensified. Specifically, when applying jet CPAP with a PEEP of 10 mbar, the noise level exceeded 80 dB, irrespective of whether prongs or a mask were used. Detailed values are given in Table 1.

**Table 1:** Sound levels according to modality as well as level of non-invasive respiratory support.

Level of support	2	4	6	8	10	12
Jet CPAP prongs (mbar)	74 dB_SPL_	77 dB_SPL_	78 dB_SPL_	79 dB_SPL_	83 dB_SPL_	83 dB_SPL_
Jet CPAP mask (mbar)	73 dB_SPL_	76 dB_SPL_	78 dB_SPL_	80 dB_SPL_	81 dB_SPL_	84 dB_SPL_
HFNC (L/min)	70 dB_SPL_	69 dB_SPL_	70 dB_SPL_	71 dB_SPL_	73 dB_SPL_	75 dB_SPL_

CPAP, continuous positive airway pressure; HFNC, high-flow nasal cannula.

### Invasive ventilation (HFOV)

Overall, non-invasive respiratory support produced higher noise levels than invasive ventilation via HFOV, which generated an average noise level of 69 ± 1 dB. However, a significant difference in noise levels was only observed between invasive ventilation (HFOV) and jet CPAP, regardless of whether prongs or a mask were used (Table 2).

**Table 2:** Statistical differences in sound level between the different modalities of respiratory support and ventilation.

Modality 1 (m1)	Modality 2 (m2)	Mean m1 (dB_SPL_)	Mean m2 (dB_SPL_)	Mean difference	*p*-values	95% confidence interval	
						Lower bound	Upper bound
Jet CPAP prongs	Jet CPAP mask	79	78,667	−0,3330	0,8790	−5,1010	4,4340
Jet CPAP mask	HFNC	78,667	71,333	7,3330	0,0030	3,2520	11,4150
Jet CPAP prongs	HFNC	79	71,333	7,6670	0,0010	3,8650	11,4680
HFOV	Jet CPAP prongs	69	79	−9,6000	<,001	−13343,0	−5,8570
HFOV	Jet CPAP mask	69	78,667	−9,2670	<,001	−13,3640	−5,1690
HFOV	HFNC	69	71,333	−1,9330	0,1170	−4,4560	0,5900

CPAP, continuous positive airway pressure; HFNC, high-flow nasal cannula; HFOV, high-frequency oscillatory ventilation.

### Third-octave band spectral analysis

Frequency analysis revealed a peak within the high-frequency band, particularly in regard of non-invasive respiratory support, located within the third-octave frequency bands ranging from 4,000 to 8,000 Hz. Notably, this phenomenon was particularly pronounced with the use of jet CPAP, whether with prongs or a mask. In contrast, the use of HFNC showed a significantly reduced peak in this frequency range. Furthermore, when using jet CPAP via a mask or prongs, a secondary, albeit smaller, peak was found around 1,000 Hz.

**Figure 1:**
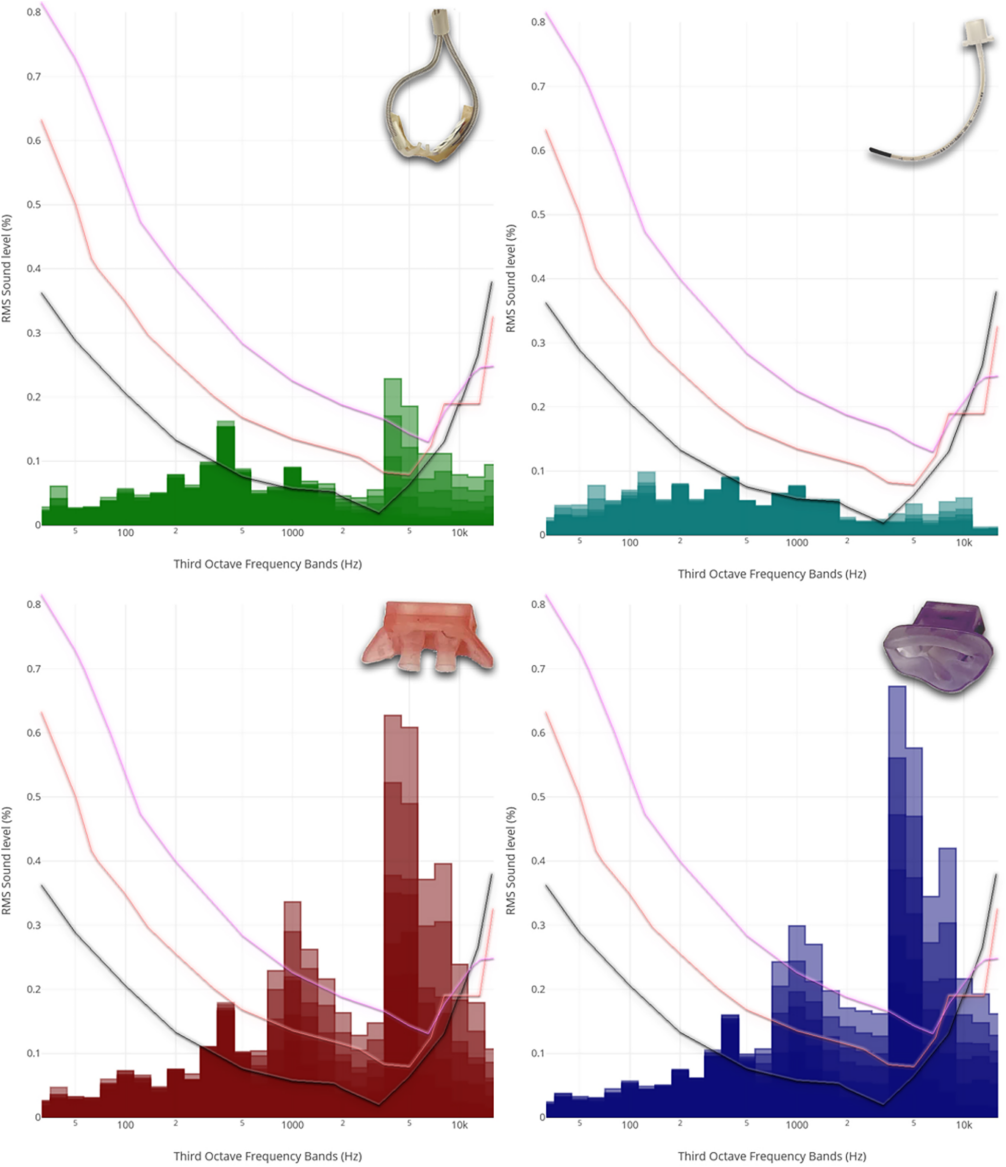
Third-octave band analyzed noise levels of the different respiratory support modalities. From left to right, from top to bottom: Third-octave band analyzed noise levels of **HFNC** (2, 4, 6, 8, 10 and 12 L/min), **HFOV** via **endotracheal tube** (MAP: 8, 12 and 15 mbar; amplitude: 20, 40 and 60 mbar), jet **CPAP prongs** (2, 4, 6, 8, 10 and 12 mbar), and jet **CPAP mask** (2, 4, 6, 8, 10 and 12 mbar). For the visualization, the respective curves were superimposed and presented together. Each curve refers to an **adult hearing threshold (grey)**, a term **newborn hearing threshold (orange)** and a 30 weeks **preterm infant hearing threshold (purple)** (hearing thresholds have been added according to Lasky and Williams 2005). Interactive application with all audio examples: https://muwidb.univie.ac.at/respiration/.

As shown in Figure 1, jet CPAP produced a peak within the frequency range exceeding 5,000 Hz, which coincides precisely with the auditory threshold of neonates and preterm infants.

Within the third-octave bands between 3,000 and 8,000 Hz, there was a linear increase in sound energy with ventilation intensity in the context of non-invasive respiratory support. This increase was much less pronounced for HFNC compared to jet CPAP. Regarding various settings of HFOV, including amplitudes of 20–60 mbar and mean airway pressures of 8–15 mbar, sound energy levels within the third-octave band remained relatively consistent.

### Acoustic camera recordings

**Figure 2:**
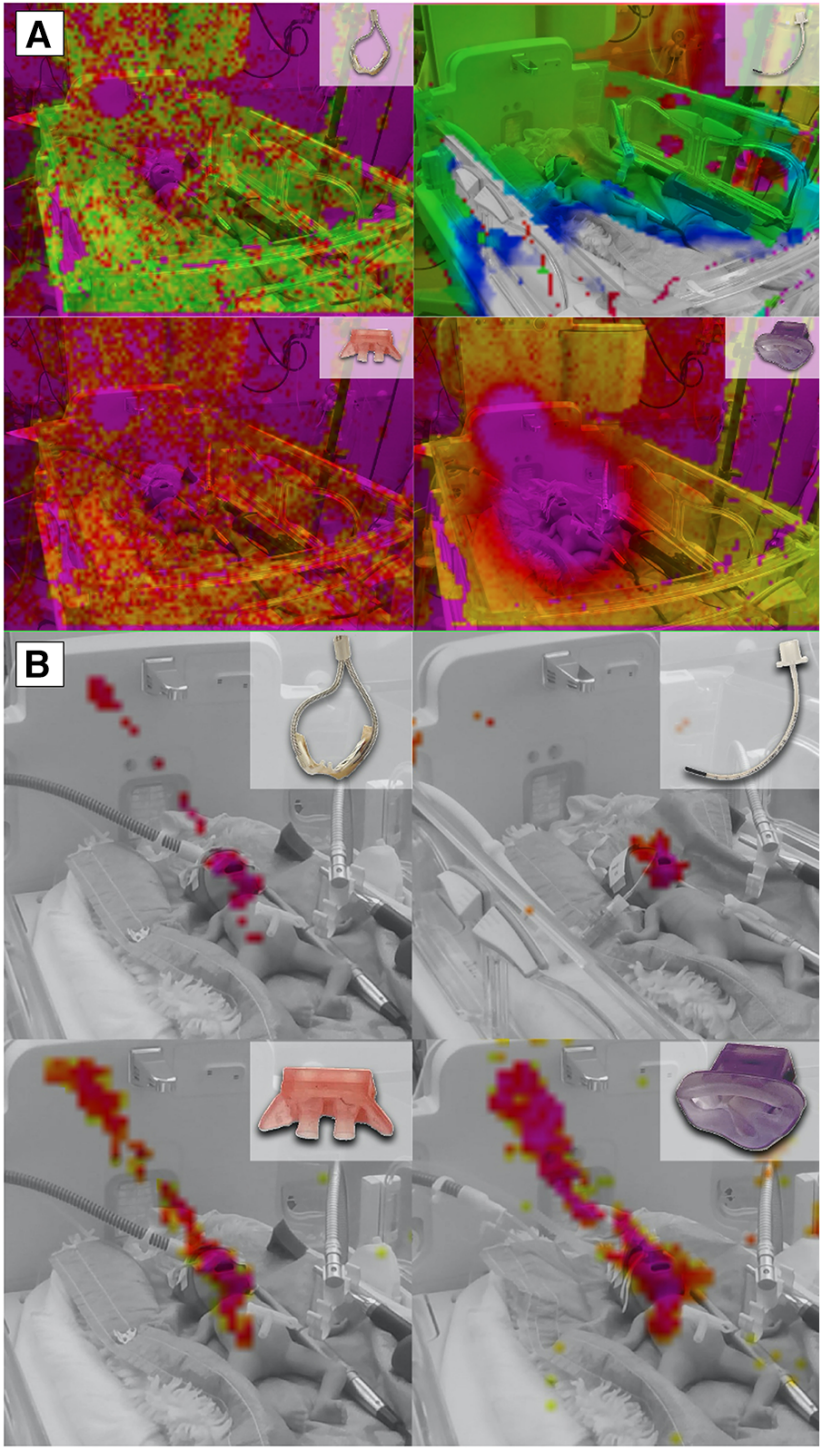
Acoustic Camera Recordings of different respiratory support modalities. (**A**) Acoustic camera recordings of respiratory support with HFCN at 12 L/min, jet CPAP at 12 mbar, and HFOV at MAP: 12 mbar and amplitude 60 mbar, respectively, showing the sound radiation in levels from 0 dB to −60 dB (violet = loudest via red—yellow—green to blue = quietest) and below (no colour); (**B**) Acoustic camera recording of respiratory support with HFCN at 12 L/min, jet CPAP at 12 mbar, and HFOV at 12 mbar and 60 mbar, respectively, showing the loudest parts of the image (purple to yellow, range 10 dB) in the frequency range of 5,000–7,200 Hz.

This observation became also evident in the recordings obtained with the acoustic camera. A comparison of different modes of respiratory support revealed a distinct discrepancy, with HFNC and HFOV showing lower acoustic emissions compared to jet CPAP (Figure 2A).

When the focus of the acoustic camera was directed towards the frequency range corresponding to the most sensitive auditory range of preterm infants (5–7.2 kHz), a comparison showed a notable difference between the various respiratory support modalities (Figure 2B). Specifically, the jet CPAP devices was significantly greater within this specific frequency range compared to the other respiratory support modalities.

### Sound characteristics

As sound is not just a combination of noise level (dB_SPL_) and frequency (Hz), further measurements were taken in order to provide an objective description of the sound characteristics.

**Figure 3:**
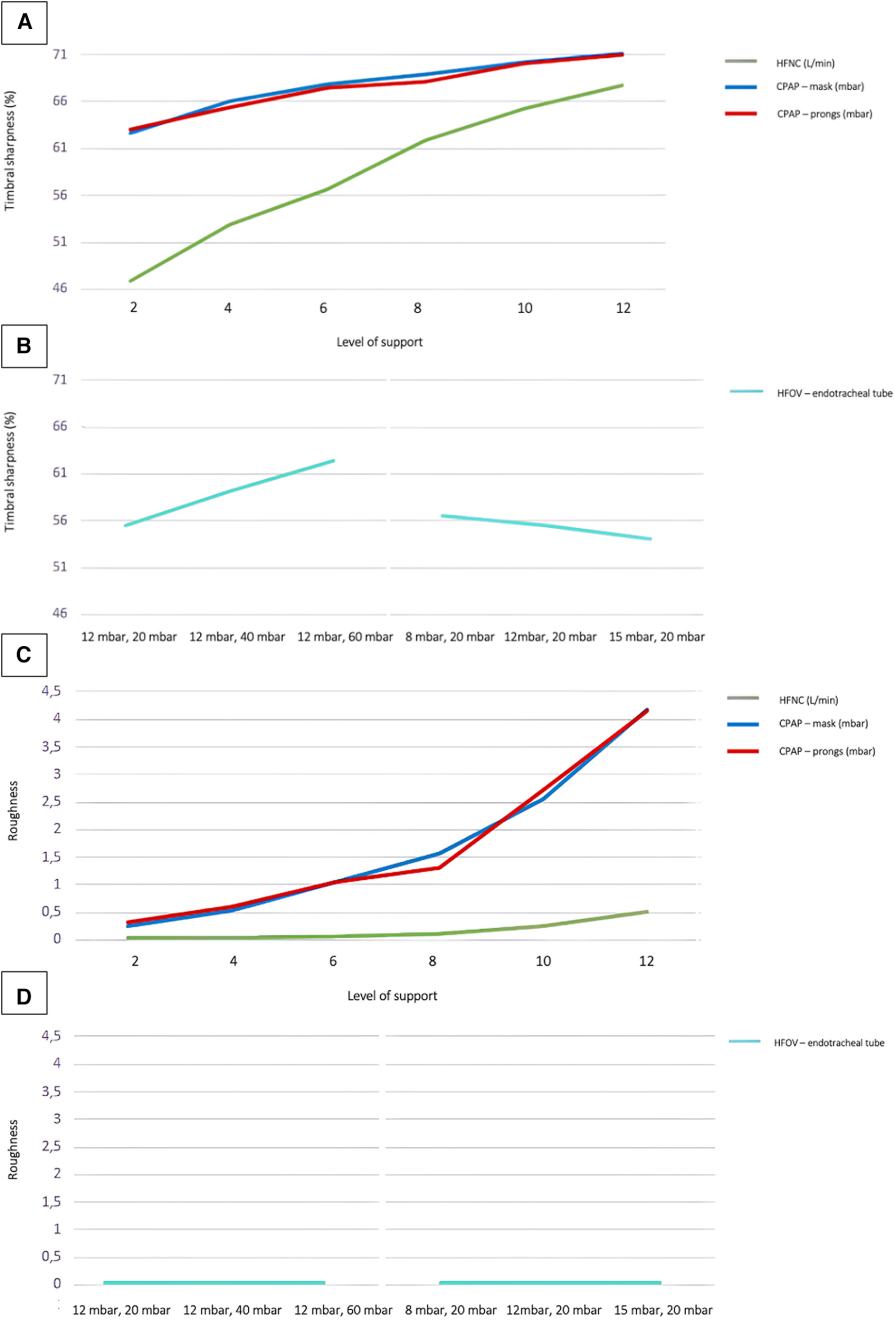
Timbral sharpness and roughness of different respiratory support modalities. Timbral sharpness with increasing ventilation intensity in non-invasive (**A**) and invasive (**B**) respiratory support. Roughness (Sethare)s with increasing ventilation intensity in non-invasive (**C**) and invasive (**D**) respiratory support. Regarding the sound level values of HFOV the measurements were done at different MAP and amplitude levels: 12 mbar/20 mbar; 12 mbar/40 mbar; 12 mbar/60 mbar; 8 mbar/20 mbar; 12 mbar/20 mbar; 15 mbar/20 mbar.

Increased respiratory support not only influenced noise levels and frequencies, but also manifested in timbral attributes. Particularly parameters associated with timbral brightness, such as spectral centroid, spectral roll-off, spectral entropy, spectral decrease, zero crossing rate, brightness, high-frequency content, and sharpness were affected. Figure 3 (A + B) shows a sharpness curve with increasing support as pars pro toto: Clearly, the increase in sharpness was most pronounced when using jet CPAP and exhibit a direct correlation with the level of support provided. Notably, for HFOV, the modulation of sharpness depended primarily on the amplitude, ranging from 20 to 60 mbar, rather than on the mean airway pressure, ranging from 8 to 15 mbar.

When using jet CPAP, particularly at PEEP levels above 8 mbar, timbral characteristics such as roughness (as defined by Sethares or Vassilakis), spectral flux, and other subtle modulatory elements, became more pronounced with elevated levels of support. In contrast, measurements on HFNC and HFOV showed almost no timbral roughness, even under conditions of high levels of support.

### Inside vs. outside the incubator

**Figure 4:**
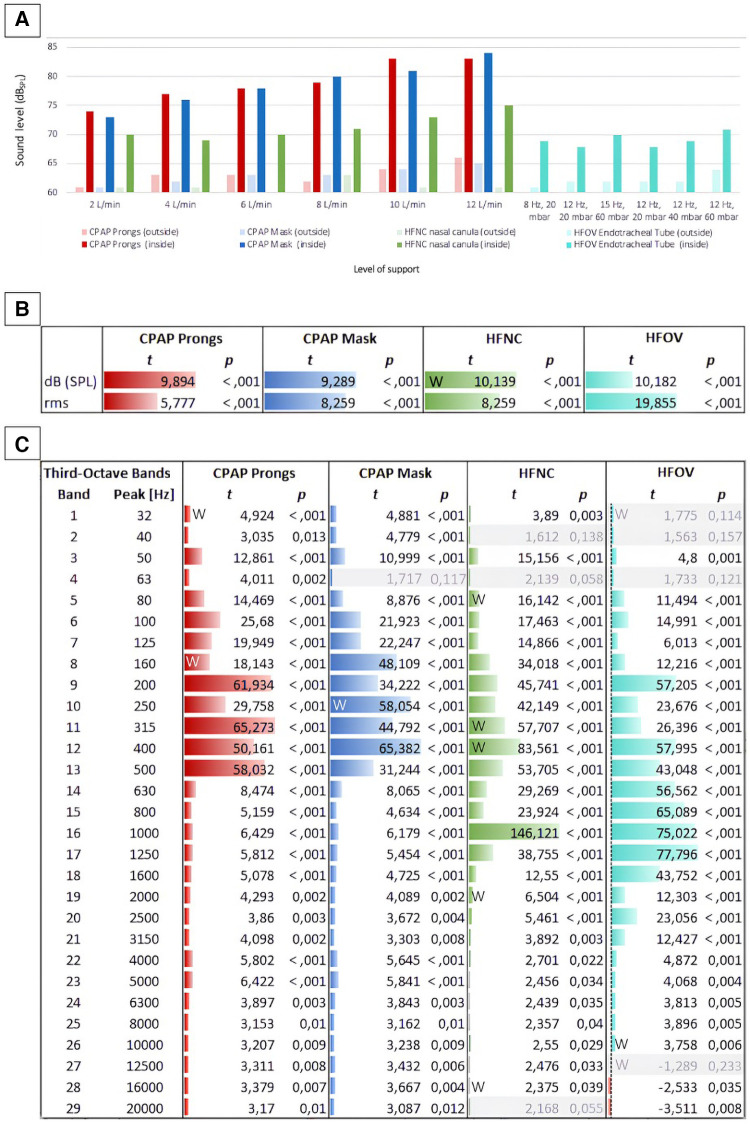
Sound levels inside and outside the incubator concerning different modes of respiratory support. (**A**) Shows a comparison of all measured levels (in dBSPL): The bars depict sound level inside (saturated colors) and outside (light colors) the incubator at different intensities of non-invasive respiratory support (jet CPAP, HFNC) and HFOV. (**B**) Shows the differences between the levels (inside vs. outside) per respiratory device as t values of a *t*-test. For each respiration device, all levels inside the incubator are compared with all levels outside the incubator. Both the differences between the sound levels (dBSPL Sound Pressure Level) and between their effective values (rms, root mean square) are shown in t values. While dBSPL describes the peak level of an audio signal, rms (root mean square) describes the average power level of an audio signal. This means that rms with a time window of 300 ms corresponds better to the temporal resolution of the human auditory perception for levels, as short-term level changes lower than 300 ms are less detectable here. (**C**) Shows the differences between the 1/3rd octave band filtered levels (in the incubator vs. outside) per respiration device as t values. For grayed out rows, *p* is larger than 0.05, i.e., in this frequency band the level differences between inside and outside are not significant enough. Data that were not normally distributed were marked with a W.

The incubator shields against noise. However, it does not only reduce the noise level from the outside to the inside but also vice versa, as shown in Figure 4A. Recordings outside the incubator uniformly resulted in lower noise levels across all varieties of respiratory support compared to the corresponding measurements inside the incubator.

While the acoustic levels outside the incubator did not exceed 66 dB_SPL,_ even during high levels of respiratory support, the recordings next to the ear of the mannequin inside the incubator peaked at 84 dB_SPL_ (Figure 4B).

The differences between the measurements inside and outside the incubator were evident not only in terms of sound levels but also in the sound characteristics.

A comparison of timbral attributes inside and outside the incubator during various respiratory support modalities revealed expected variations, particularly within the lower frequency bands. Using jet CPAP devices, frequency components between 80 and 500 Hz were significantly amplified inside the incubator. Conversely, using HFNC or HFOV, timbral differences between inside and outside the incubator were particularly pronounced in the mid-frequency range from 200 to 1,600 Hz. A particularly significant difference was observed at 1,000 Hz using HFNC, with the sound level recorded inside the incubator being considerably elevated compared to the measurements outside the incubator.

The *t*-test analyses for various respiratory modalities further revealed that respiratory support noises sounded brighter inside than outside the incubator, attributed to the effective sound attenuation capabilities of the incubator walls.

A similar observation was made with regard to the occurrence of timbral booming within the incubator, although this phenomenon was particularly observed during jet CPAP usage. This can be attributed to the jet CPAP sound, which boasts low-frequency components exciting the natural resonances of the incubator.

A detailed analysis of timbral components, specifically focusing on aspects like roughness, noisiness, spectral fluctuations, and the proportion of percussive elements in the sound, showed that, predictably, the manifestations of roughness, spectral flux, and percussive noise components were significantly more pronounced inside the incubator compared to outside (Supplementary Figure S1). This observation was particularly true when considering the roughness exhibited by the two jet CPAP modalities.

## Discussion

The primary objective of this study was to characterize the sounds generated by common respiratory support and invasive ventilation devices inside an incubator used in neonatology. Gaining a deeper understanding of the sound dynamics of these devices is crucial, given their frequent use during the first weeks of life of preterm infants. In this study we used the FABIAN HFOI® as ventilator, and therefore all presented results refer to this equipment and might be different with other devices.

The acoustic landscape in the neonatal intensive care unit depends primarily on medical staff and medical equipment. In the case of medical equipment, devices for mechanical ventilation and non-invasive respiratory support are major contributors, as the sound intensity increases up to 100 dB when they are in use (26, 27).

Our results confirm the previously reported elevated noise levels in the incubator caused by respiratory support devices, by far exceeding recommended NICU noise levels of 45 dB. A considerable decrease of the noise level with gradual reduction of the level of support is reported for all types of respiratory devices in our study.

The jet CPAP resulted to be the loudest non-invasive respiratory support. Its noise level was confirmed in previous studies (28, 29). It is important to notice that for this study we used only the Infant Flow® equipment as CPAP system, which even if used with sound absorber, has been reported to be one of the loudest devices compared to other CPAP systems (e.g.,: Baby Flow®), which demonstrated to be up to 30 dB quieter than the one we have used in this study (29).

Our data show a significant difference in noise levels between jet CPAP and HFNC.

In the realm of neonatal care, the choice between CPAP and HFNC is critical. Both are non-invasive respiratory support technologies that play a pivotal role in managing neonatal respiratory distress syndrome, a common complication in premature infants (30).

CPAP has long been established as a conventional method of support (31). Its mechanism involves delivering a steady stream of air or oxygen to maintain airways open, thereby facilitating better gas exchange. However, the complexity of CPAP administration demands high proficiency from medical staff. Improper use can lead to some undesirable effects such as nasal mucosal injury, nasal granuloma, vestibular stenosis, septum deformation or deletion, besides causing discomfort due to the necessity of special caps for fixation (30).

On the other hand, HFNC emerges as an alternative technology offering several benefits over CPAP. It delivers a blend of air and oxygen through a nasal cannula at high flow rates, which can result in improved gas exchange, promotion of alveolar dilation, protection of airway mucosa, and a reduction in the work of breathing. The gentler nature of HFNC, along with its ease of use and reduced risk of nasal injury, positions it as an attractive alternative for neonatal respiratory support (30).

The literature comparing the two modalities presents a complex picture. Some studies suggest HFNC is as effective as CPAP as primary approach to mild to moderate respiratory distress syndrome in preterm infants older than 28 weeks of gestation (32). Other research, however, indicates a higher failure rate for HFNC, pointing to CPAP as a more reliable option for certain clinical scenarios (33).

This discrepancy underscores the need for careful consideration of patient-specific factors and conditions when choosing between CPAP and HFNC.

From an acoustic perspective, if clinically possible, an early switch from a jet CPAP device to HFNC is preferred, if the devices used are the same as in this study. However, it should be considered that even though sound levels generated by the HFNC are lower than those generated by jet CPAP, they are still above the recommended threshold by the AAP.

The discrepancy in noise values between CPAP and HFNC as showed in our results are in line with what has been showed by Surenthiran and colleagues (28), where noise intensities in the post-nasal space in those receiving CPAP support were higher than in the other groups, reaching mean levels of up to 102 dB_SPL_, and increased with increasing flow rates. In contrast, Koenig et al. (34) concluded that the use of HFNC resulted in higher sound levels compared to CPAP, using the Fisher & Paykel Nasal High Flow™ system and Vapotherm Precision Flow® for HFNC support and the Dräger Babylog® 8,000 for CPAP, respectively. Measurements in the external auditory meatus of infants done by Roberts et al. (35) showed no significant differences in the average noise levels generated by HFNC and bubble CPAP. Regarding HFOV, Goldstein et al. (36) reported that the sound level varied depending on the different neonatal ventilator models used.

Lastly, the cumulative impact of noise, also influenced by the bone conduction, should not be underestimated. However, there are limited information on this topic. We can only assume that the noise sensation increases in such a case; therefore, an adequate application of the device is of high importance. In the context of this topic, improvements involve the utilization of padding materials and ensuring a secure fixation of the respiratory support.

As the sound source is located inside the incubator, it is important to note that the noise level outside the incubator—and thus as heard by the NICU staff and parents—is not as loud as it is for the infant inside the incubator. Our studýs acoustic camera recordings further revealed that the noise emanated not just from the different respiratory devices, but there was also a significant sound reflection from the head wall of the incubator. This could potentially be reduced by altering the incubator's design or, if feasible, by installing a broadband absorber following hygiene standards.

As previously shown in the work of Reuter et al. (21), it is evident that the incubator has a significant timbral influence on the sound transmission from inside to outside (as shown in Supplementary Figure S2). This is particularly evident in the case of sound features that describe timbral brightness, such as spectral centroid, spectral roll-off, spectral spread, spectral bandwidth, the zero-crossing rate.

Preterm birth is associated with a disruption of the normal auditory development and perception. The extrauterine hearing exposure in the NICU setting differs significantly from the intrauterine hearing experience: Although intrauterine measurements have also shown sound level peaks close to 90 dB (37), external sounds are attenuated by the uterus and the amniotic fluid, creating a vastly distinct auditory environment dominated by low frequency sounds not exceeding 30 dB (11, 38). In contrast, our data from recordings inside the incubator show a peak in the high frequency band around 5,000 Hz with the use of non-invasive respiratory support, which is particularly unphysiological in this context. Exposure to elevated noise levels in the NICU can lead to various adverse effects in preterm infants, including stress responses, altered physiological stability, sleep deprivation, and changes in the autonomic, metabolic and endocrine system (19).

It has been known since the 1980s that the auditory threshold of newborns differs significantly from that of adults. Whereas adults typically demonstrate increased sensitivity in the 2–4 kHz frequency range, peaking at around 2.7 kHz (39), newborns exhibit a distinct auditory sensitivity within the 5.1–7.2 kHz range (13, 40–43). Notably, this sensitivity profile evolves as the individual matures. Up to the age of 2 years, the peak of auditory sensitivity gradually shifts to around 3 kHz. It then undergoes a gradual transition to 2.75 kHz by the age of 7 years, which is comparable to the hearing threshold of adults (41, 44, 45).

This shift in hearing sensitivity can be attributed to the growth of the outer ear canal. At the time of birth, the length of the outer ear canal is approximately 11–17 mm. However, by the time an individual reaches adulthood, it extends to a length of 27–32 mm (42, 44, 46, 47).

The natural resonance of the almost cylindrical outer ear canal, sealed at one end by the eardrum, can be calculated using the formula: Resonance frequency = (speed of sound, roughly 343 m/s at 20°C)/(4 times the length of the outer ear canal). Hence, the calculated theoretical natural resonance frequency of the outer ear canal at birth is in the range of 5,044 Hz (at a length of 17 mm) to 7,795 Hz (at a length of 11 mm). In adulthood, the theoretical natural resonance frequency of the outer ear canal ranges from 2,680 Hz (at a length of 32 mm) to 3,176 Hz (at a length of 27 mm). Importantly, premature infants are likely to have even shorter outer ear canals, resulting in even higher predicted natural resonance frequencies. In the womb, the embryo is surrounded by amniotic fluid, rendering the natural resonance of the ear canal almost negligible due to the significantly faster speed of sound in water, approximating 1,484 m/s at 20°C. Sound waves traveling at such a high speed are predicted to have shorter wavelengths, which in turn could result in even higher theoretical resonance frequencies. At this speed, calculated resonance frequencies in the range of 33,727 Hz (at a length of 11 mm) and 21,824 Hz (at a length of 27 mm) are well above the limits of human hearing. In essence, the amniotic fluid of the womb abrogates the resonant frequency contribution of the outer ear canal to increased hearing sensitivity. Once exposed to an air environment after birth, high frequency sounds generated by respiratory support devices can reach the particularly sensitive hearing threshold range of preterm infants over a prolonged period.

Very and extremely preterm infants often spend weeks to months in the NICU and often require long-term mechanical ventilation or non-invasive respiratory support. This in turn leads to a long-term exposure to unphysiological soundscapes, assuming that the auditory system is functional already by 25 weeks of gestation. This time of gestation until 5–6 months of corrected age is a crucial period for the auditory development. During this period, the hair cells of the inner ear establish connections with underlying neuroanatomic structures and fine-tune their responsiveness to specific frequencies and intensities. Throughout this period, the auditory system requires active stimulation, including speech, music and environmental sounds, to facilitate its development.

It seems that preterm infants are unable to discriminate meaningful sounds when exposed to noise levels exceeding 60 dB (10). The data of our study indicate that noise levels generated by any of the respiratory support and ventilation modalities tested surpass this threshold, even at the lowest support settings. This may potentially contribute to a higher risk of adverse hearing and language development outcomes in preterm infants. In fact, prior research data from our group revealed significant deficits in the ability of preterm infants to discriminate speech from non-speech at term equivalent age. This suggests potential alterations in the development of functional neural networks crucial for language acquisition (9) we postulate that these disparities in speech discrimination between preterm and term infants may, in part, be attributable to variations in the early auditory experiences of these infants.

## Limitations

The study has limitations that should be taken into consideration when interpreting the study's findings: (1) All recordings were conducted at the Pediatric Simulation Center of the Comprehensive Center of Pediatrics. Despite our efforts to simulate a realistic environment closely resembling our NICU and employing actual NICU equipment, the recordings are inherently influenced by the dimensions of the simulation room and placement of the microphones. (2) It is important to acknowledge that the use of a mannequin may introduce variations in sound that differ from those produced by a real human body. (3) Another limitation was that our investigation exclusively focused on HFOV within the spectrum of invasive ventilation modes. In the clinical setting, conventional invasive ventilation, including assist-control modes, stands as another important practice that is widely used in the neonatal setting.

## Conclusion

In summary, despite the AAP recommendation of noise levels not exceeding 45 dB during the day and 35 dB at night in NICUs, the use of respiratory support and ventilation devices alone consistently exceeded these levels in our study.

In contrast to the predominantly low-frequency sound environment *in utero*, the use of respiratory support leads to peaks in the high-frequency range, which is considered to be particularly unphysiological.

Intriguingly, the protective effect of the incubator appears to have an inverse impact, with the perception of noise levels from respiratory support and ventilation devices being lower for NICU staff and parents outside than for the infant inside the incubator.

Both jet CPAP via a mask and jet CPAP via prongs exhibited significantly higher sound levels compared to HFNC and to HFOV delivered through an endotracheal tube. Moreover, HFNC and HFOV showed a reduced acoustic presence compared to jet CPAP within the frequency range where the preterm infant's hearing is at its most sensitive. Therefore, it is reasonable to speculate that the potential for auditory impairment and challenges in language acquisition may be more pronounced in preterm infants who require long-term jet CPAP therapy during their time in the incubator. Overall, this study bears the potential to support a deeper understanding of sound dynamics in the NICU setting.

## Data Availability

The raw data supporting the conclusions of this article will be made available by the authors, without undue reservation.
