# Supplementary material for: “Every breath you take”: evaluating sound levels and acoustic characteristics of various neonatal respiratory support and ventilation modalities
Source: Front Pediatr. 2024 Apr 19;12:1379249. doi: 10.3389/fped.2024.1379249 (PMC11066168; doi:10.3389/fped.2024.1379249)
Supplement: Supplementary file 1 [file Datasheet1.pdf]

Every Breath you take

Supplementary material

Figure-S1 Differences between timbre/brightness features inside and outside the incubator (t-test) with different respiratory support devices

	CPAP Prongs		CPAP Mask		HFNC		HFOV	
	t	*p*	*t*	*p*	*t*	*p*	*t*	*p*
Spectral Centroid	-3,525	0,005	-2,083	0,064	-2,065	0,066	-17,122	< ,001
Spectral Kurtosis	4,24	0,002	2,53	0,03	2,377	0,039	11,648	< ,001
Spectral Rolloff	-5,803	< ,001	-3,194	0,01	-2,708	0,022	-33,224	< ,001
Spectral Skewness	5,056	< ,001	2,962	0,014	2,849	0,017	9,688	< ,001
Spectral Spread	-6,091	< ,001	-4,453	0,001	-6,275	< ,001	-32,038	< ,001
Spectral Bandwidth	-5,989	< ,001	-4,366	0,001	-5,847	< ,001	-47,136	< ,001
Spectral Contrast: 0-200 Hz	1,28	0,229	-4,726	< ,001	-7,603	< ,001	0,475	0,647
Spectral Contrast: 200-400 Hz	0,746	0,473	-2,211	0,051	-0,738	0,478	1,389	0,202
Spectral Contrast: 400-800 Hz	4,434	0,001	2,368	0,039	13,658	< ,001	-0,292	0,778
Spectral Contrast: 800-1600 Hz	-16,321	< ,001	-15,181	< ,001	-24,686	< ,001	-14,917	< ,001
Spectral Contrast: 1600-3200 Hz	2,005	0,073	-1,949	0,08	2,514	0,031	4,327	0,003
Spectral Contrast: 3200-6400 Hz	8,14	< ,001	5,711	< ,001	11,118	< ,001	15,389	< ,001
Spectral Contrast: 400-1600 Hz	-7,542	< ,001	-7,675	< ,001	-11,065	< ,001	-9,074	< ,001
Timbral Booming	-4,567	0,002	-4,523	0,002	NaN		NaN	
Zero Crossing Rate	0,851	0,415	1,418	0,186	-0,017	0,987	-6,856	< ,001

Figure-S2 Differences between modulation/noisiness features inside and outside the incubator (t-test) with different respiratory support devices

	CPAP Prongs		CPAP Mask		HFNC		HFOV	
	t	*p*	*t*	*p*	*t*	*p*	*t*	*p*
Inharmonicity	-3,931	0,003	-2,733	0,021	-2,789	0,019	-2,252	0,054
Roughness (Sethares)	2,822	0,018	2,804	0,019	2,182	0,054	9,675	< ,001
Roughness (Vassilakis)	2,863	0,017	3,061	0,012	1,56	0,15	-3,206	0,013
Spectral Entropy	-2,934	0,015	-1,755	0,11	-1,548	0,153	-8,802	< ,001
Spectral Flatness	-6,666	< ,001	-4,634	< ,001	-3,048	0,012	-28,328	< ,001
Spectral Flux	5,883	< ,001	5,408	< ,001	7,879	< ,001	14,639	< ,001
Spectral Flux (> 1000 Hz)	5,338	< ,001	4,986	< ,001	3,805	0,003	7,992	< ,001
Spectral Flux (< 100 Hz)	10,562	< ,001	8,069	< ,001	17,955	< ,001	3,761	0,006
Harmonic Energy	6,096	< ,001	5,668	< ,001	9,003	< ,001	15,309	< ,001
Percussive Energy	5,379	< ,001	4,939	< ,001	7,129	< ,001	17,295	< ,001
Harmonic Percussive Ratio	-3,704	0,004	-1,782	0,105	-4,558	0,001	-3,243	0,012
